# A high-performance sub-THz planar antenna array for THz sensing and imaging applications

**DOI:** 10.1038/s41598-024-68010-9

**Published:** 2024-07-24

**Authors:** Muhammad Zubair, Abdul Jabbar, Farooq A. Tahir, Jalil ur Rehman Kazim, Masood Ur Rehman, Muhammad Imran, Bo Liu, Qammer H. Abbasi

**Affiliations:** 1 James Watt School of Engineering, University of Glasgow, Glasgow, G12 8QQ UK; 2 School of Electrical Engineering and Computer Science, National University of Sciences and Technology, Islamabad, Pakistan; 3 Artificial Intelligence Research Centre, Ajman University, Ajman, UAE

**Keywords:** Engineering, Electrical and electronic engineering

## Abstract

Terahertz (THz) spectral region from 0.1 to 3 THz is envisaged to hold immense potential in the next generation of wireless technologies. Recently, research has focused on this terahertz gap, because of its unprecedented channel capacities. At the physical layer, the design complexities and fabrication of THz devices, especially antennas are the prime bottlenecks to realize its full potential. This article introduces a cost-effective, easy-to-fabricate, and reproducible sub-THz antenna design based on a single-layer planar printed circuit board technology. The antenna incorporates carefully designed quasi-cross slots and applied machine learning-assisted global optimization techniques to achieve the desired performance metrics. The antenna performance is elucidated through numerical simulations and verified through a rigorous in-house THz experimental framework around 100–110 GHz. The proposed antenna offers a peak gain of 13.90 dBi with less than 1 dB variation within the entire band of 100–110 GHz. The antenna holds the potential to achieve terabits per second data rates and futuristic high-resolution short-range THz imaging applications.

## Introduction

The next-generation wireless technologies for communication and sensing require ultra-reliability, extremely low latency, a very high data rate, and fine-grain spatial differentiation. To meet these challenges, recent advancements in THz technology have urged interest in the 100 GHz to 10 THz frequency bands, known for high-resolution imaging, ultra-fast wireless communication, and precise sensing^1^. Frequencies between 100 and 300 GHz, often termed "sub-THz," serve as a crucial bridge between microwaves and infrared light, characterized by its broadband, unregulated bandwidth, non-ionizing nature, and high-resolution capabilities^2,3^. These features open up unprecedented opportunities for diverse applications in THz communications, security screening, non-destructive testing (NDT), material characterization, quality control, THz imaging and sensing^4,5^.

THz waves possess unique properties that could potentially support data rates hundreds of times faster than current wireless technologies. These advancements could pave the way for applications such as ultra-fast wireless internet, high-definition streaming, and real-time, high-resolution imaging. However, despite these promising applications, the effective utilization of the THz frequencies is impeded by several challenges, including propagation characteristics, networking protocols at higher layers, and the efficient design of physical layer (PHY) devices^6^. More specifically, the realization of THz communication faces numerous challenges, particularly in the design of suitable antennas. Moreover, the THz antenna is one of the most important components and it plays a significant role in both impedance matching and power source. Challenges include the development of efficient radiators, ease of integration with THz devices, and low fabrication complexity to achieve reliable communication links^32^. Addressing these challenges can unlock the full potential of THz communication and harness its transformative capabilities for future wireless systems. In response to these challenges, various THz antenna design types have been explored, including on-chip antennas (AoC)^33^, sub-THz horn antennas, dielectric resonator antennas, and microstrip patch antennas. Among these, silicon-based on-chip antennas experience high radiation losses and low gain due to the high permittivity and low resistivity of the silicon substrate^7^. On the other hand, horn antennas are bulky and expensive while facing miniaturization challenges, thereby making it difficult to incorporate them into planar circuits^8^. Meanwhile, dielectric resonator antennas, known for their compact size and high radiation efficiency, encounter difficulties in achieving wide bandwidth and robust mechanical designs^9^. These issues with THz antenna design critically impact the viability and effectiveness of THz systems for use in imaging and sensing applications.

Among the various antenna designs, microstrip patch antennas stand out for their potential to address some of these challenges, albeit with their limitations in terms of bandwidth and gain. These antennas benefit from their size, low-profile planar configuration, and manufacturing simplicity using printed circuit board (PCB) technology on low-cost substrates. However, these conventional antennas are limited by a very narrow frequency band, often just a fraction of a percent^10^. Various approaches have been employed to enhance the bandwidth, such as incorporating various slots in the patch radiator designs to yield extra controllable resonances ^11^^,^^12^. Furthermore, to achieve a higher gain, the employment of an array configuration is often necessary. For example, several PCB-based THz antennas designed for high gain and small form factors have been presented in the literature ^13–16^. However, achieving a wide impedance bandwidth and high gain over a wide frequency band is quite challenging with a single-layer PCB antenna. In^13^, a 1 × 5 series-fed 0.1 THz antenna is designed on a liquid crystal polymer (LCP) substrate to achieve a maximum gain and radiation efficiency of 15.82 dBi and 52%, respectively. However, its suboptimal dimensions for integration present challenges for THz applications where compact and high-performance antennas are required. Similarly, in^14^ 1 × 3 element array was designed with a resonant frequency of 312 GHz. Due to the very short wavelength (0.03–3 mm) at THz frequencies, fabricating THz antennas with acceptable tolerances is challenging, especially when attempting to use cost-effective fabrication processes. Moreover, in^15^, a 1 × 3 microstrip patch single-layer PCB antenna array is designed to cover the 99–101.5 GHz band. However, its fractional bandwidth is less than 2 GHz. Adding to this discussion^16^, emphasizes a multi-layer PCB-based sub-THz 4 × 1 AiP array, which gives a fractional bandwidth of 13% and enhanced gain. However, the complexity of its structure is high.

Our design integrates quasi-crossslot features to tackle the distinct challenges posed by the THz frequency range, where minor dimensional variations have a pronounced impact due to ultra-high frequencies and small wavelengths. This strategy aims to achieve an optimal balance between compactness and high-performance radiation characteristics, alleviating the size constraints. The antenna improvements encompass bandwidth, gain, radiation pattern, and impedance matching throughout the operational bandwidth, all of which are crucial for achieving superior performance in the demanding THz range. Transitioning to an antenna array, we recognize that achieving high-gain antennas often necessitates designing an array configuration. However, designing an efficient wideband power divider (feed network) for these arrays poses a significant challenge, particularly in the THz range where ultra-high frequencies and small wavelengths magnify the impact of even minor changes in the feed junction lengths. Such precision requirements in design and fabrication underscore the complexities of optimizing antenna performance for the sub-THz band, where a delicate balance of size, gain, bandwidth, radiation efficiency, and side lobe level must be maintained.

This paper addresses these challenges by employing advanced design and optimization techniques to achieve a cost-effective, compact and high-performance THz antenna array. The Surrogate-Assisted Differential Evolution Algorithm for antenna synthesis (SADEA-I)^19–21^ has been employed to find the optimal design parameters to satisfy the specifications. SADEA-I offers 3 to 10 times optimization efficiency, improvements, and higher optimization ability compared to several popular antenna optimizers^22,23^. Its key features include the differential evolution (DE) algorithm as the global search engine, the Gaussian process for surrogate modeling, and a surrogate model-aware evolutionary search framework for effective model management^24^. The SADEA-I optimized antenna array shows competitive performance in terms of gain, bandwidth, and radiation efficiency, along with effective control of sidelobe levels and low susceptibility to fabrication tolerances.

## Antenna design

### The unit element design

**Figure 1:**
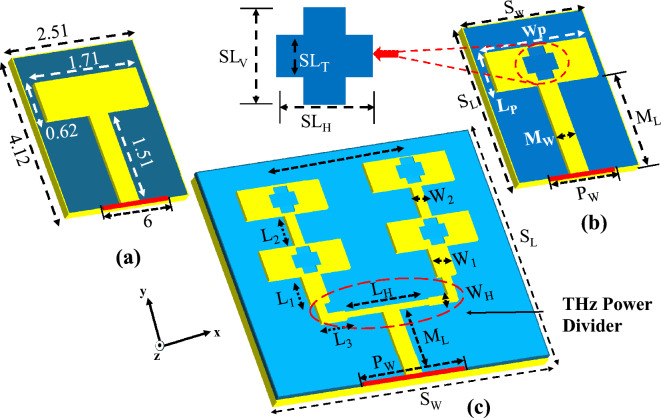
Design evolution and geometrical dimensions of the proposed sub-THz antenna.

The first step involves the design of a rectangular patch antenna with a dimension of 1.71 mm × 0.60 mm as shown in Fig. 1a, aiming the resonant frequency of 105 GHz using the standard design equations. The antenna is designed on RT/Duroid 5880 substrate with a dielectric constant of 2.2 with a thickness of 0.125 mm and a dissipation factor of 0.004, and a copper thickness of 17.5 $\mu m$.

The specific dimensions of the antenna were optimized to achieve a balance between the antenna's resonant frequency and maintain its compact size, ensuring compatibility with compact THz systems. The antenna is modeled in CST Microwave Studio (CST-MWS 2022) using the finite integration technique (FIT) method with an accuracy of − 40 dB and a maximum cell density of 20 cells per wavelength, resulting in approximately 3,344,156 hexahedral mesh cells in total. The antenna design comprises a driven rectangular patch incorporating a slot. These slots are strategically positioned at the head and center to create a quasi-cross-slot configuration as shown in Fig. 1b. This is particularly important given that patch antennas inherently possess narrowband. Enhancing the impedance bandwidth, one approach is to increase the substrate thickness and reduce the quality factor. However, this can lead to increased antenna dimensions and losses, along with altered radiation patterns due to stronger surface waves, which are undesirable in the sub-THz band. To maintain simplicity in the sub-THz band design and reduce complexities, quasi-cross slots were incorporated into the radiating patches, a technique proven effective in literature for modifying current paths and generating higher-order current modes. Such modifications lead to notable improvements in the antenna's performance, including reduced side-lobe levels, increased gain, and correction of the squint effect, thereby overall enhancing the radiation characteristics. Furthermore, the antenna's bottom side is covered with a full copper layer, serving as a ground plane to permit broadside radiation beams.

### Hybrid 2 × 2 array design

Designing antenna arrays is crucial for enhancing THz system performance, especially for achieving high antenna gain. Our design process commenced by evaluating various parameters sensitive to optimization performance within a corporate-fed network as shown in Fig. 1c. The designed feeding network, based on transmission line theories, precisely controls the amplitude and phase of the current supplied to each antenna element, ensuring effective power distribution. The feed junctions and transitions are carefully matched using λ_0_/4 transmission line sections to minimize reflections, where even minor discrepancies in dimensions can substantially affect the array's bandwidth and performance—key considerations for THz sensing and imaging applications that demand exceptional precision and reliability.

A hybrid parallel-series fed array configuration was employed to achieve a compact size and high gain beam. The inter-element element gap was optimized at 1.32 mm (0.68 λg or 0.46 λ_0_), as demonstrated in Fig. 1. Where λg and λ_0_ represent the guided and free space wavelengths, respectively, at 105 GHz. This optimum spacing was determined using the SADEA-I algorithm, which aims to minimize mutual coupling and improve radiation efficiency, thus enhancing bandwidth and array gain to address the significant path loss challenges at sub-THz (100–110 GHz) frequencies.

To ensure desired impedance matching across the target frequency band, precise adjustments to the feed line dimensions, including width and length, to achieve impedance levels of 50Ω, 70Ω, and 100Ω. Concurrently, the array's symmetrical layout around the y-axis plays a significant role, not only in improving the uniformity of the radiation pattern but also in simplifying the fabrication process. Through extensive simulations, involving over 550 iterations with the SADEA-I algorithm, we established the optimal dimensions for a high-performance array topology, showcasing the critical role of AI-driven techniques in achieving stringent design criteria in THz antenna arrays.

### Geometric optimization and performance enhancement

The design of the proposed antenna presents significant challenges, primarily due to its small form factor and the need for high gain and directivity in the sub-THz band. Additionally, the requirement for the antenna to maintain a broad beamwidth—to facilitate detection from multiple angles in THz imaging—further complicates the design. This requires maintaining a relatively consistent boresight gain across the desired bandwidth, which leads to the need for geometrical optimization in the slotted patch as well as in the feeding structure to optimize overall performance within the target band.

To obtain the optimal antenna design, the Surrogate-Assisted Differential Evolution Algorithm for Antenna Synthesis (SADEA-I)^19^ is employed via the MATLAB Antenna Toolbox. SADEA-I is the first generation of the SADEA algorithm series^19–21,34,35^. SADEAs combine supervised learning and evolutionary search techniques to effectively explore and exploit the antenna design search space. In SADEA-I, the supervised learning method used is Gaussian process (GP), which constructs surrogate models that approximate antenna performances. The surrogate modeling works harmoniously with the differential evolution (DE)-based global search for a balanced exploration and exploitation of the design space. The model management method used in SADEA-I is the surrogate model-aware evolutionary search framework^24^.

In each iteration, a surrogate model is built using the available simulated candidate designs and their performances. New candidate designs are generated by DE search operators, and their performances are predicted by the GP surrogate model. Using the prediction results, the predicted best candidate design is simulated and used to update the surrogate model for the next iteration. This iterative process continues until the optimal design is obtained. More details about SADEA-I are in^19^.

Widely utilized local optimization techniques (e.g., Trust Region Framework (TRF) in CST Microwave Studio) were applied, but the results were far from satisfactory; although the specifications for bore-sight gain and total efficiency were met, the $\text{max}\left(\left|{S}_{11}\right|\right)$ is poor (i.e., about − 6 dB). Moreover, the use of available global optimization techniques (e.g., particle swarm optimization), was estimated to require a prohibitive amount of time without a guarantee of success^19,35,36^. Consequently, as described, the SADEA-I from the MATLAB Antenna Toolbox was used to obtain an optimal design. The initial phase involved element design, with design variables detailed in Tables 1, 2.

**Table 1:** Search ranges and dimensions of the single antenna element (dimensions in mm).

S.No	Parameter	Lower bounds	Upper bounds	SADEA-I optimum
1	Patch length (L_p_)	0.512	0.95	0.70
2	Patch width (W_p_)	1.61	3.50	1.71
3	Microstrip length (M_L_)	1.23	3.00	2.12
4	Feed Guide width (P_W_)	6.00	10.00	6.95
5	Microstrip width	0.20	0.35	0.31
6	Width of Slot (SL_W_)	0.20	0.32	0.21
7	Length of Slot (SL_L_)	≥ SL_w_	0.33	0.22
8	Vertical Slot depth (SL_V_)	0.38	0.46	0.38
9	Horizontal Slot depth (SL_V_)	≥ SL_V_	0.46	0.47
10	Substrate Length (SL) = M_L_ + (2 × SL_h_) + 0.2

**Table 2:** Search ranges and dimensions of the antenna array (dimensions in mm).

S.No	Parameter	Lower bounds	Upper bounds	SADEA-I optimum
1	Horizontal line length (L_H_)	0.33	4.00	3.56
2	Horizontal line width (W_H_)	0.20	0.28	0.20
3	Microstrip length (M_L_)	1.25	3.00	2.15
4	Feed line1 length (L_1_)	0.40	0.90	0.88
5	Feed line1 width (W_2_)	0.32	0.46	0.33
6	Feed line2 length (L_2_)	0.60	0.90	0.78
7	Feed line2 width (W_2_)	0.20	0.25	0.22
8	Substrate Length (S_L_) = M_L_ + W_H_ (1.5 × (L_1_ + W_2_) + 0.2			

The objective function is to minimize the fitness function $({F}_{\text{mpa}})$ in (1). This ensures that the antenna meets the target performance metrics in Table 3. While the overall size of the antenna is not directly specified in (1), the defined search ranges and geometric constraints are deliberately chosen to promote a compact, low-profile design. For instance, during the optimization, the parameter (S_**Lv**_) must be greater than (S_**Lh**_) to maintain a minimal physical footprint which is among the main design goals.

**Table 3:** Performance summary for the proposed sub-THz antenna configuration.

Configuration	Design constraints	Specifications	SADEA-I optimum
Single Element with Quais-Cross Slots	S_11_	≤ − 10 dB	− 12 dB
	Realized Gain	≥ 6 dBi	8.2 dBi
	Total Efficiency	≥ 70%	79%
2 × 2 Array	S_11_	≤ − 10 dB	− 11 dB
	Realized Gain	≥ 10 dBi	12.9 dBi
	Total Efficiency	≥ 70%	82.2%
	SLL (Φ = 90°)	≤ − 15 dBi	− 15.12 dBi
	SLL (Φ = 0°)	≤ − 10 dBi	− 10.21 dBi

1 $${F}_{\text{mpa}}=\text{max}\left(\left|{S}_{11}\right|\right)+w\cdot \text{max}\left(\left[6\text{ dBi}-{G}_{\text{min}},0\right]\right)+w\cdot \text{max}\left(\left[0.70-{\eta }_{\text{min}},0\right]\right)$$

Here, the worst performances of $\left|{S}_{11}\right|$, bore-sight gain and total efficiency are used, which are at different frequencies. In this equation, w denotes the penalty coefficient, preset to a value of 50, which is set empirically^22^. Instead of using a weight-sum of the three performance metrics, for which, the balance of them is hard to control, specifications are provided for bore-sight gain and total efficiency. This setting ensures that violations of bore-sight gain and total efficiency specifications, denoted by ${G}_{\text{min}}$ and ${\eta }_{\text{min}}$, are significantly penalized, prioritizing these aspects in the optimization process. After conducting 350 electromagnetic (EM) simulations, the specifications were met within a total design time of approximately 10.5 h.

Using the individual elements, the array configuration is then optimized. This phase addresses the arrangement and collective behaviour of the antenna elements to achieve desired array-level characteristics such as broadside gain, and minimal sidelobes in the radiation pattern. In high-frequency (THz) regimes, the efficiency of microstrip lines drops significantly because of serious losses at the bends and the discontinuities. As a result, developing a low-loss, planar feeding network is imperative for maintaining efficient performance.

SADEA-I investigates various array configurations, including the spacing between elements, distribution patterns, and feeding strategies. The design parameters, along with their respective search ranges as detailed in Table 2. These parameters are crucial in reducing the fitness function, denoted as ${(F}_{mpa})$ in (2), to meet the requirements. Even though the size of the antenna is not explicitly included in (2), the defined search parameters and geometric limitations are strategically set to guarantee a compact and low-profile design. Thus, as part of the optimization process, the key performance metrics and design constraints are encapsulated in the fitness function ${(F}_{mpa})$ defined as follows:

2 $${F}_{mpa}=max(|S\_11 |)+w\cdot max([10dBi-{G}_{\text{min}},0])+w\cdot max([0.70-{\eta }_{\text{min}},0])+w.max(\left[-15dBi-S{L}_{\text{max}(\Phi =90^\circ )},0\right]+w.max(\left[-10dBi-S{L}_{\text{max}(\Phi =0)^\circ },0\right])$$

**Figure 2:**
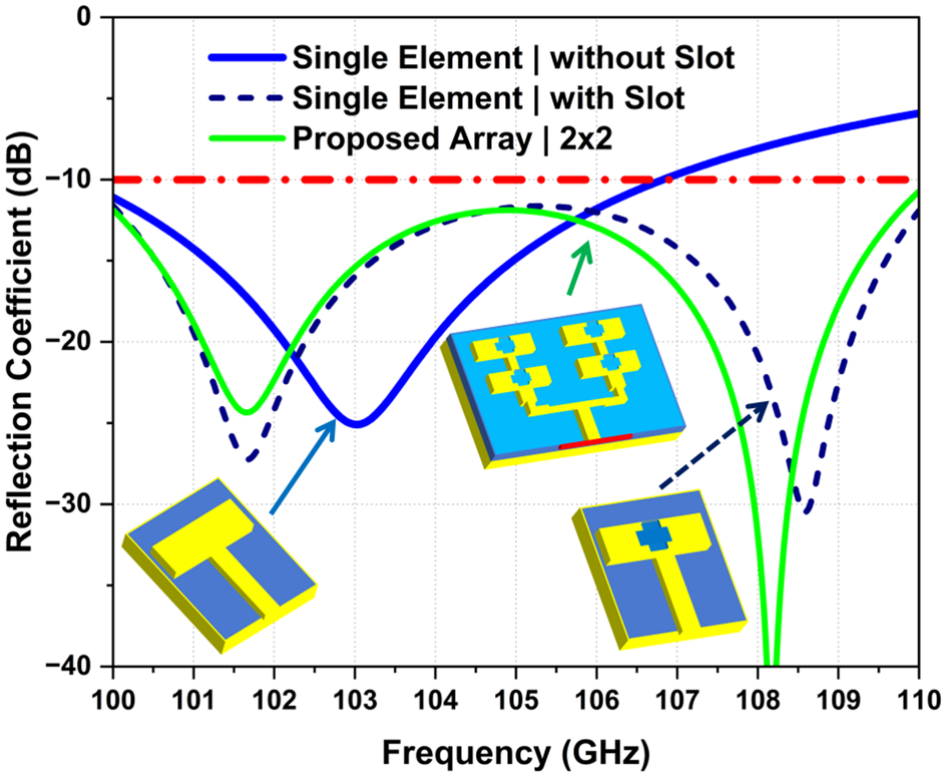
Simulated reflection coefficient of various antenna design steps.

**Figure 3:**
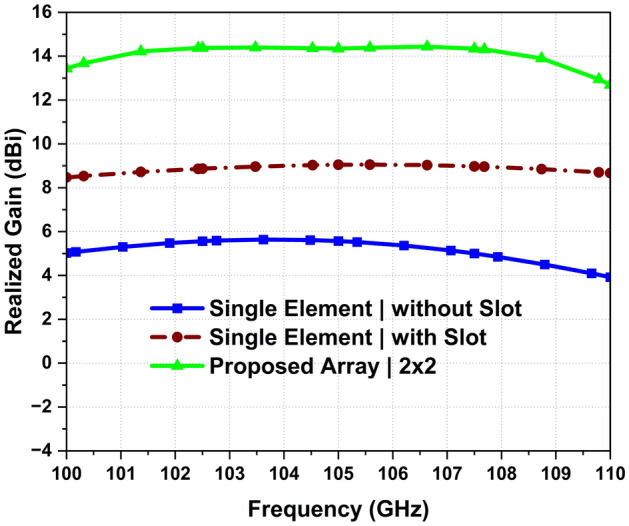
Simulated realized gain.

**Figure 4:**
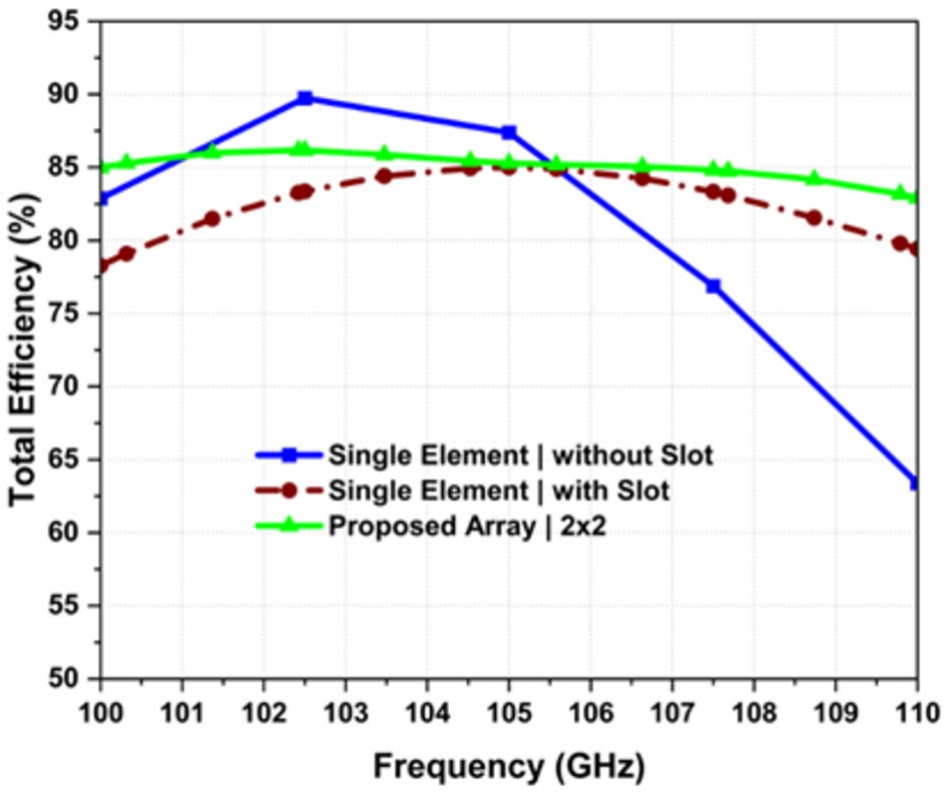
Simulated efficiency.

After 550 EM simulations, an optimal design was obtained. The overall optimization time was approximately 21.5 h. The simulated S-parameters, shown in Fig. 2, demonstrate that the reflection coefficient meets the requirements.

for the desired band (100–110 GHz) and the mutual coupling is maintained below − 10 dB and − 15 dB in the plane (phi 0°) and (phi 90) respectively as shown in Fig. 6. The boreside gain and total efficiency are depicted in Figs. 3 and 4, respectively. The physical implementation is illustrated in Fig. 8. The dimensions of the entire array are approximately 5.2 mm × 5.12 mm × 0.127 mm which corresponds to 1.82 λ_0_ × 1.79 λ_0_ × 0.044 λ_0,_ where λ_0_ is the free space wavelength at 105 GHz.

### Simulated results and discussion

For the single-element design, we initially excluded the quasi-cross slots to establish a baseline performance. The time-domain (TD) simulation in the full-wave electromagnetic CST solver was performed. The simulated reflection coefficient of a single element without quasi-cross slots shows a reflection coefficient below 10 dB, and provides a narrow bandwidth of about 3.5 GHz (101–104.5 GHz), as illustrated in Fig. 2. Upon the integration of a quasi-cross slot into the single-element antenna, the modification led to a significant redshift, causing the antenna to resonate dually at approximately 100.8 GHz and 108.4 GHz, with a return loss of less than 10 dB. To further enhance performance, the SADEA-I algorithm was used as discussed in the previous section. This adjustment expanded the -10 dB impedance bandwidth to cover a 10 GHz range, from 100 to 110 GHz. These modifications had an impact on the current distribution and impedance characteristics of the antenna.

The motivation for extending array design, extensively discussed in the antenna design section, was to examine the potential for additional performance improvements. Following the same optimization strategy, the 4-element array not only maintained wideband performance but also showed improvement in impedance bandwidth to 10 GHz (100–110 GHz), as depicted in Fig. 2, while maintaining the dual resonances, occurring at 100.8 GHz and 108.1 GHz, with a return loss of 25 dB and 40 dB, respectively. These resonances show a slight shift toward lower frequencies, due to structure modification. This adjustment leads to a wideband response and more consistent operational bandwidth, essential for advanced sub-THz imaging and sensing applications^25^.

Moreover, Fig. 3 illustrates a systematic comparison of the realized gain for the various antenna design configurations. A single antenna element without slots achieved a maximum gain of 5.65 dBi at 103.62 GHz and less than 6 dBi throughout the desired band (100–110 GHz). However, the incorporation of a primitive quasi-cross slot enhances the boresight gain by 2 dBi at certain frequencies within in operational band. After an extensive optimization using the SADEA-I algorithm, the maximum boresight gain of the single element with the quasi-cross slot increased to approximately 9.04 dBi at 105.5 GHz, resulting in an improvement of roughly 2.5 dBi. Moving to the array configurations, the 4-element array displays a significant uplift in the gain, reaching a maximum of approximately 14.43 dBi at 105 GHz, while the minimum in-band realized gain is observed to be 12.90 dBi. In contrast, the simulated realized gain consistently exceeds 13.1 dBi across the band of interest (100–110 GHz). The gain variation within the operating band of interest for this array is minimal, suggesting a stable performance across the spectrum with variations possibly within a narrow window of less than 1 dBi. These findings underscore the impact of design and optimization strategies on enhancing the performance of antenna elements and their configurations.

For the single element with quasi-cross slots across the entire band of interest, the optimized minimum In-band total efficiency is 79.21%, as illustrated in Fig. 4. It's important to note that total efficiency represents the ratio of radiated power to input stimulated power, accounting for losses due to mismatch. Similarly, the same optimization technique was employed for the 4-element array design; the minimum in-band total efficiency is 82.2% and this performance is consistently maintained above the threshold across the entire frequency range, showcasing a stable efficiency profile.

**Figure 5:**
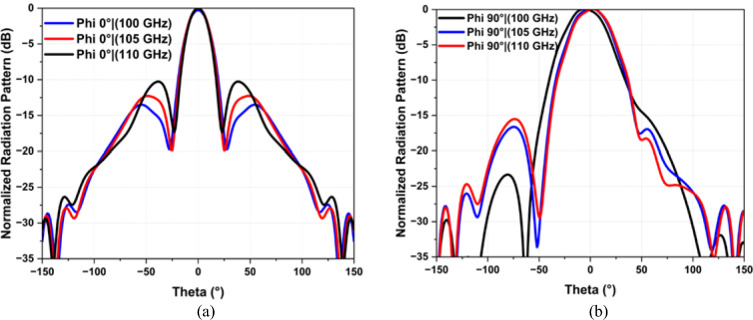
Simulated radiation patterns at 100, 105, 110 GHz (**a**) phi 0° and (**b**) phi 90°.

**Figure 6:**
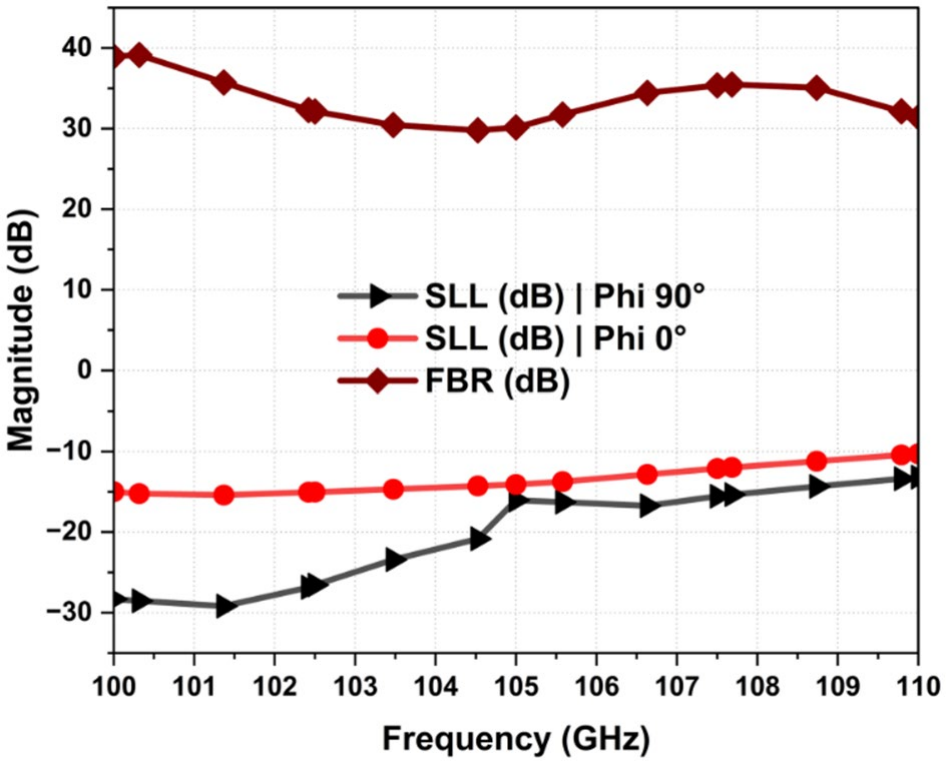
SLL | phi 0° and phi 90° and Front-to-Back Ratio of the Proposed Array Antenna (**a**) phi 0° and (**b**) phi 90°.

The 2D radiation patterns in the x–z plane (phi 0°) and y–z plane (phi 90°) at 100, 105, and 110 GHz are depicted in Fig. 5a and b, respectively. These patterns exhibit high stability across the frequency range. In the x–z plane (phi 0°), the half-power beamwidth (HPBW) are 23.12°, 21.5°, and 20.2°, while the side-lobe levels (SLLs) are − 14.2 dB, − 12.3 dB, and − 10.21 dB at 100 GHz, 105 GHz, and 110 GHz, respectively, as shown in Fig. 6.

Similarly, in the y–z plane (phi 90°), the HPBW are 39.7°, 38.6°, and 37.5°, with SLLs of − 26.2 dB, − 16.3 dB, and − 13.1 dB at 100 GHz, 105 GHz, and 110 GHz, respectively, as shown in Fig. 5b. In the y–z plane (phi 90°), the half-power beam width (HPBW) are relatively higher, due to the presence of a fan-shaped pattern, which is expected because of the linear array configuration.

Notably, the side lobe levels (SLLs) maintain a value below − 10 dB in both planes, indicating minimal cross-polarization levels in the direction of the main beam, as detailed in Fig. 6. Moreover, the antenna exhibits has a high front-to-back ratio, consistently exceeding 30 dB within the targeted frequency band of 100–110 GHz.

The presence of connectors can introduce distortions in the radiation pattern, primarily due to the generation of additional surface waves. To minimize this impact, a longer feed line (with proper impedance matching) can be utilized to increase sufficient distance between the connector and radiating elements. However, in real RF circuits, the connector is absent and the antenna array is directly linked to the RF circuitry.

**Figure 7:**
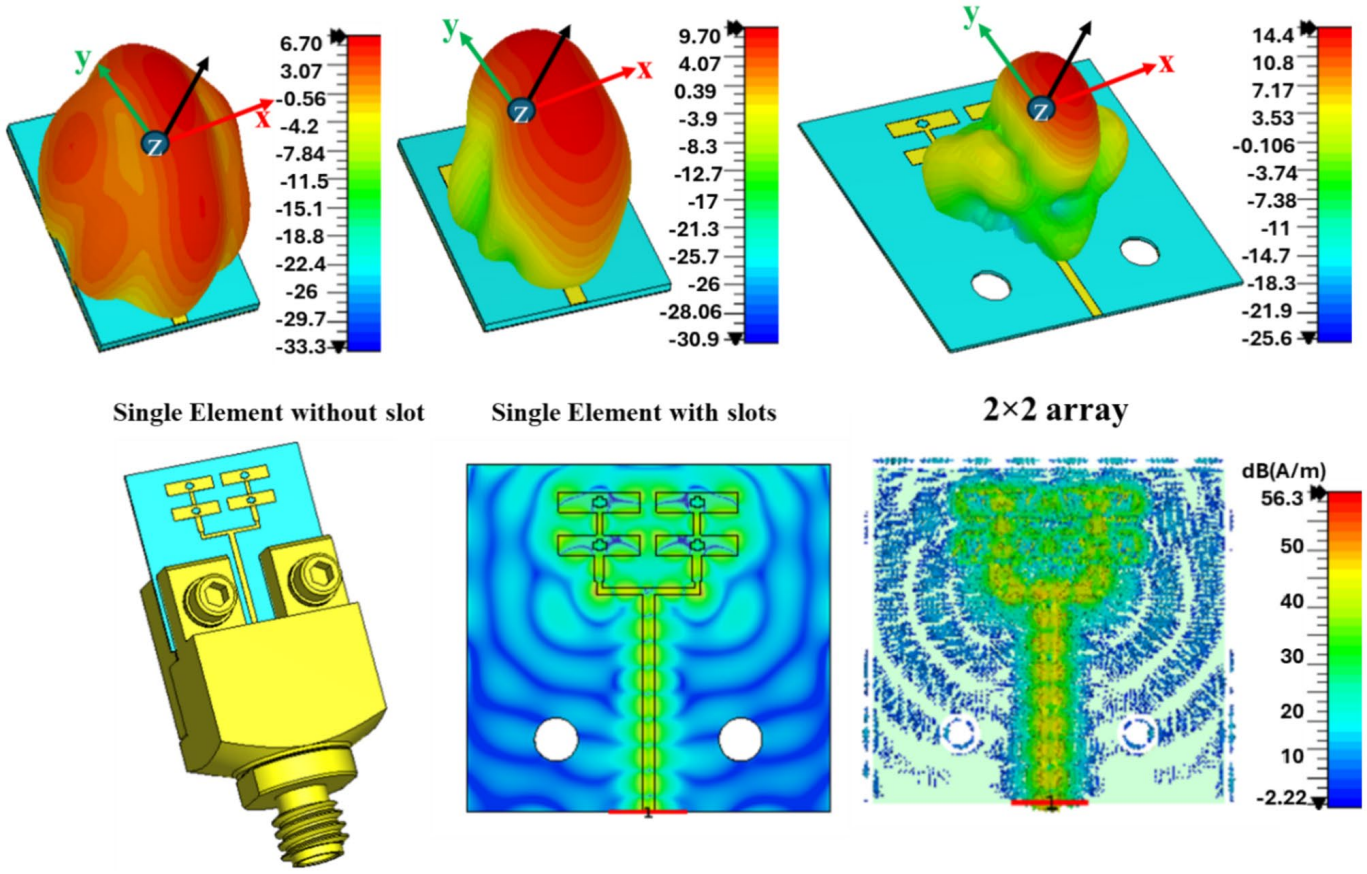
(Top) 3D radiation pattern of the proposed single element and antenna array at 105 GHz. (Bottom) Geometry of proposed array antennas with realistic connector model, E-field distribution and surface current distribution at 105 GHz.

Reflecting these practical considerations, Fig. 7 presents a visual comparison of EM properties for different antenna configurations. The top row shows the radiation pattern of the antenna elements in a 3D perspective. The single element without slots shows a relatively symmetrical radiation pattern, which is altered when a slot is integrated, indicating a change in the gain and intensity of the radiated energy. The 2 × 2 array, shows a focused a beam with pronounced main lobe, suggesting a higher gain or stronger signal in a particular direction. In the bottom left, the E-field distribution is shown with high intensity along the antenna elements, indicating effective radiating regions. The bottom right image maps the current distribution, with the most intense areas suggesting the primary radiating sections of the array. Notably, the feed network appears to be free of high-intensity current, which would correspond to unwanted radiation. This observation indicates that the array has been optimized to efficiently focus radiation through its elements efficiently, thereby achieving high radiation efficiency and potentially high gain.

### Prototype fabrication and measurement results

**Figure 8:**
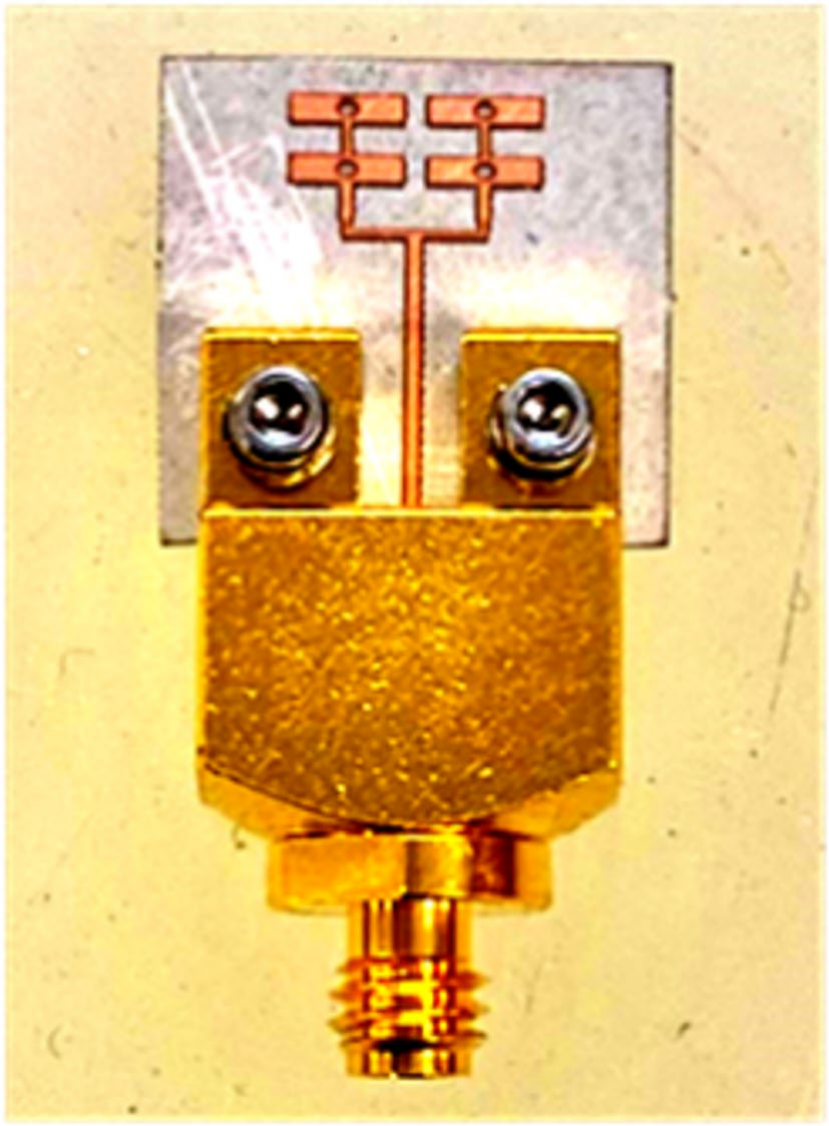
The fabricated prototype.

The sub-THz array was fabricated and tested using a low-cost standard PCB fabrication technique. For antenna array excitation, an end launch 1-mm standard solderless connector was utilized, achieving a minimal insertion loss of 0.6 dB, making it optimum for high frequencies up to 110 GHz. As soldering is not required in this type of connector, the loss due to the copper deposition during the soldering process is hence avoided (as it might adversely affect the performance at THz frequencies). It is important to note that we used 1-mm standard RF equipment in our measurement setup, which supports a maximum frequency of 110 GHz. The fabricated array, along with the integrated connector is shown in Fig. 8. The quasi-cross slots designed and fabricated have a slightly round edge rather than being purely rectangular because the slot's edge thickness is less than 0.08 mm and is quite difficult to achieve pure rectangular shapes.

For mechanical reasons and to facilitate the assembly of the connector with the array, it was necessary to extend the substrate's width to 10 mm, allowing for the placement of screw holes along the substrate's width. Moreover, a feed length of 5 mm is employed to ensure a reasonable gap between the connector and the parallel feed network so that the radiation pattern is not influenced by the connector during antenna measurements. Otherwise, as mentioned above, in practical RF circuits the antenna is typically connected directly to the RF front without the presence of a connector within the circuitry. Adding these additional dimensions led to no significant effect on radiation characteristics. However, the connector transition was also simulated in CST to verify that the connector is matched to 50 Ω.

**Figure 9:**
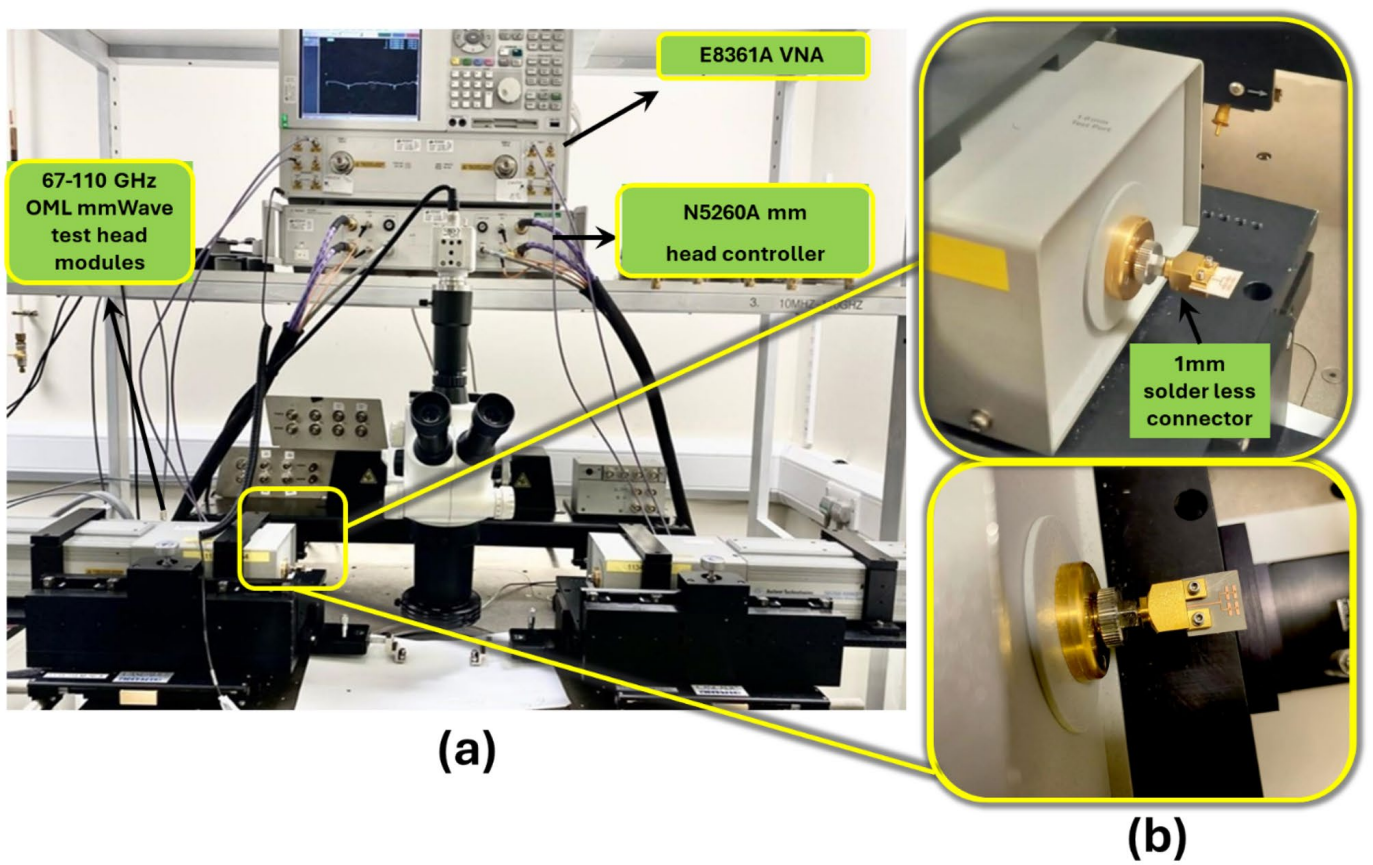
(**a**) Sub-THz antenna measurement setup using OML mmWave test head module for S_11_ measurement (**b**) Side and top view of the array connected with mmWave extender.

The measurement setup consists of an N5260A millimetre head controller connected to the Agilent E8361A Vector Network Analyzer (VNA) with a frequency range from 10 MHz–67 GHz. The head controller (N5260A) upconverts the VNA output to 110 GHz. This step ensures the accuracy and reliability of the measurement by extending the VNA capability to higher frequencies. The setup is shown in Fig. 9. The fabricated antenna is connected to the millimetre head controller through a 1 mm connector as indicated in Fig. 9a and b.

**Figure 10:**
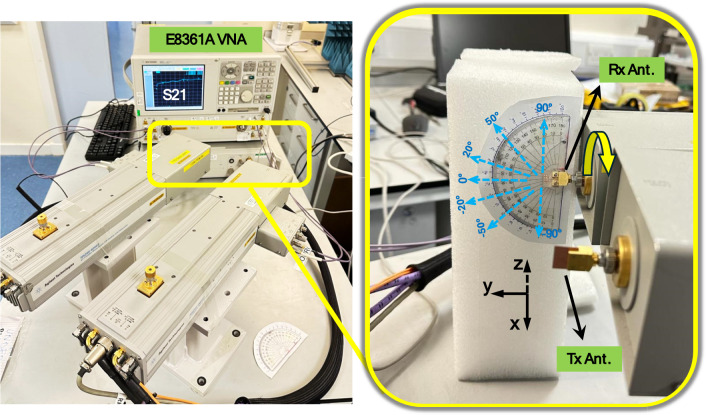
Experimental setup for radiation pattern measurement.

Following the calibration, the antenna far-field pattern measurement was conducted in a self-built environment using two identical antenna prototypes placed at a certain distance apart as shown in Fig. 10. During measurement, the transmitting and receiving antennas were positioned face to face following the E-plane or H-plane, and should satisfy the far-field condition. Only partial radiation angle and operating frequency are measured as a result of the test system limitation in the laboratory. The whole antenna performance could also be verified because the tested results agree well with the simulated ones.

The gain of the antenna array (${G}_{T}$ )was calculated by solving the Friis transmission equation as:

2 $${P}_{RX}=\frac{{P}_{TX}{G}_{RX}{G}_{TX}{\lambda }^{2}}{(4\pi R{)}^{2}}$$

3 $${G}_{T}=\frac{1}{2}\left[20{\text{log}}_{10}\left(\frac{4\pi R}{\lambda }\right)+10{\text{log}}_{10}\left(\frac{{P}_{RX}}{{P}_{TX}}\right)\right]$$

Here, ${G}_{RX}$ and ${G}_{TX}$ refers to the gain of the receiving and transmitting antenna, respectively. ${P}_{RX}$ and ${P}_{RX}$ represent the received and transmitted powers. Since the two antennas are identical, ${G}_{RX}$ is the same as ${G}_{TX}$, and both are equal to the gain of the tested antenna. The power ratio of the received and transmitted powers (${P}_{RX}$/${P}_{TX}$) is equivalent to the square of the voltage gain (S21), which was directly measured from the VNA.

**Figure 11:**
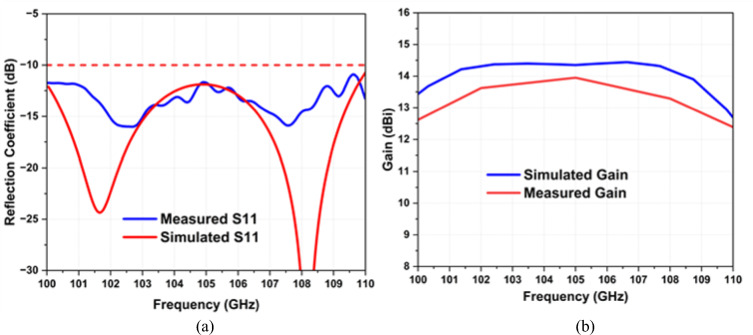
(**a**) Measured reflection coefficient. (**b**) Measured gain.

The measured operational frequency band is from 100 to 110 GHz. Both simulated and measured results are depicted in Fig. 11, illustrating a close agreement between the measured S-parameter and the simulated counterpart. Consequently, simulated and measured − 10 dB impedance bandwidths are from 100 to 110 GHz, denoting a fractional bandwidth of 9.52% as shown in Fig. 11a. A slight difference in the resonance frequency between the measured and simulated S-parameter can be attributed to fabrication tolerances or environmental factors.

The simulated and measured antenna gains are shown in Fig. 11b. It can be seen that the boresight gain versus frequency of the proposed sub-THz antenna, with a maximum gain of 13.90 dBi at 105 GHz. The measured gain remains above 12.56 dBi across the desired frequency band (100–110 GHz), showing less than 1.35 dBi variation, confirming the antenna's effectiveness for far-field THz sensing and imaging applications.

**Figure 12:**
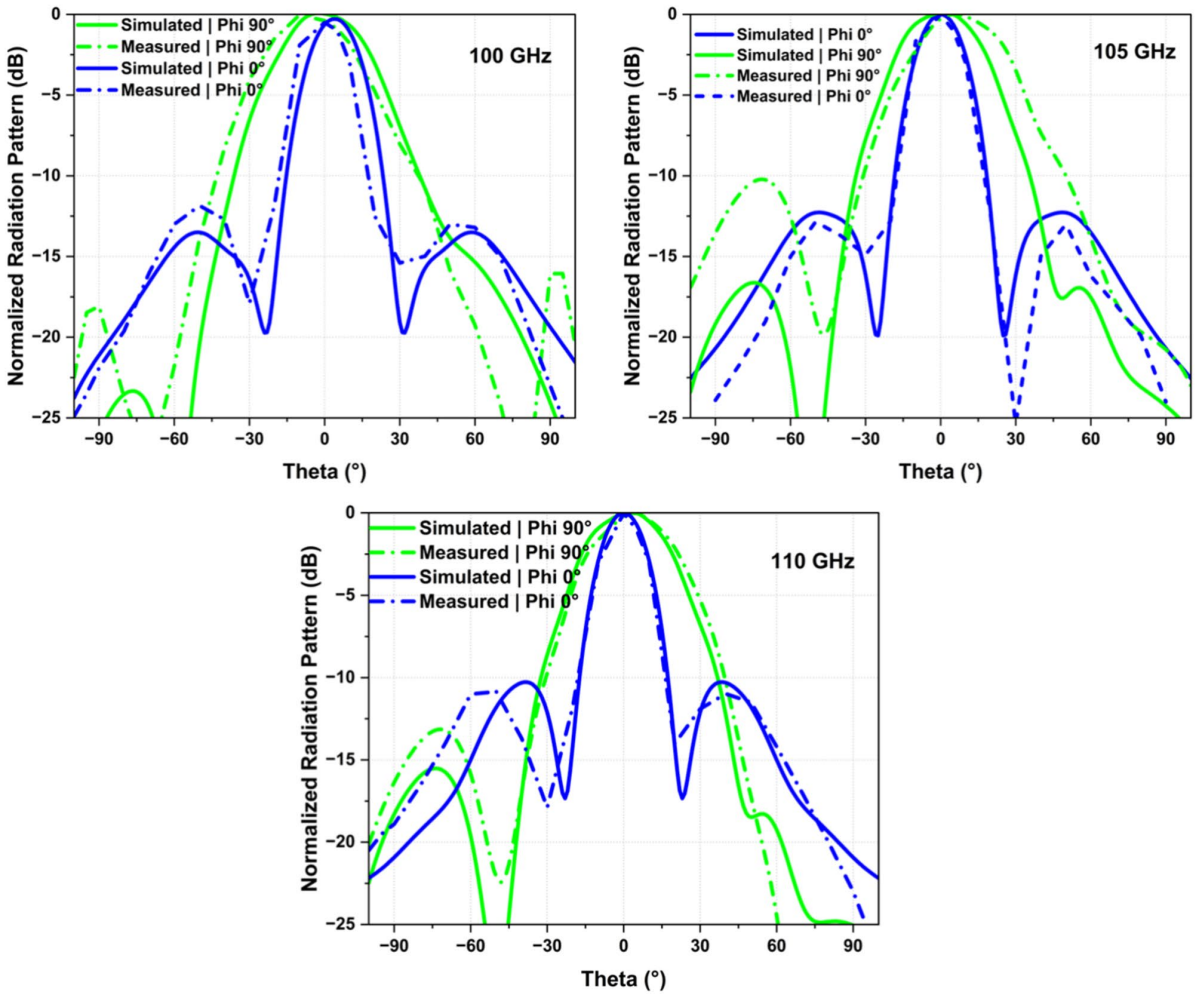
Measured radiation patterns at 100, 105 and 110 GHz.

Figure 12 illustrates the 2D radiation pattern plot for the antenna array at different frequencies (100, 105, 110 GHz), highlighting their unidirectional characteristic and stability across the operating band of interest. Moreover, The main beam angle in the phi 90° plane changes with frequency due to the progressive phase shifts across the series-fed patch elements, causing the main beam to squint or shift direction as the frequency changes. Note that this is an inherent phenomenon associated with series-fed arrays. In other words, the phase constant “β” at the antenna aperture varies with operating frequency, which results in the overall radiation pattern tilting at different frequencies. However, in the proposed design, the beam tilt in phi 90° is within 3° between 100 and 110 GHz, as shown in Fig. 12. The measured radiation pattern agrees well with the simulated one. The patterns show some discrepancies between simulation and measurement, which is common in practical antenna testing due to factors like fabrication tolerances, environmental conditions, or measurement errors. For instance, the simulated results for both Phi 0° and Phi 90° (represented by solid lines in blue and green, respectively) show smoother and more symmetrical lobe structures compared to their measured counterpart which exhibit variation and lower peak gains.

Table 4 provides a comparative analysis between the proposed single-layer planar antenna array and previously reported antenna array configuration, underscoring the distinction in performance metrics such as gain, efficiency bandwidth and design complexity. Unlike the larger and more intricate designs in the literature, our proposed antenna distinguishes itself through its compact size and efficient performance. For instance, in^13^ achieves a marginally higher gain but at the cost of a larger physical footprint and narrow bandwidth. Conversely, the designs in^28^ and^30^ are associated with lower gain. Particularly in^28^ display a again fluction exceeding 2.5 dBi within the relevant frequency range, indicating inconsistent performance across the frequency range which is suboptimal for applications requiring stable and high-quality communication.

**Table 4:** Performance comparison of the proposed antenna with other related antennas in the literature.

Refs.	Antenna type	− 10 dB imp. BW (GHz)	Peak gain (dBi) (Sim/Meas*)	Efficiency (%)	Array size (mm)	Cost	Application
^13^	Series fed array	99–101.5	15.82*	52	12 × 3.078 × 0.150	Low	Medical imaging
^14^	Microstrip patch array	304–318	11.25	90	0.706 × 0.626 × 1	–	Terahertz imaging
^15^	Series fed array	100	13.2*	–	3.078 × 2.4 × 100	Low	–
^17^	Microstrip patch array	213–0.240	16.2	–	2.81 × 2.54 × 1	–	Terahertz imaging and sensing
^18^	Substrate integrated waveguide (SIW) slot array	194–196	12.2	86	20 × 13.5 × 0.125	–	Sub-Terahertz wirless application
^26^	Planar antenna array	75–110	16/9.5*	–	7.8 × 7.8 × 0.69	High	
^27^	On-chip antenna	290–316	13.5/11.7*	≥ 55	20 × 3.5 × 0.126	High	THz application
^28^	Dipole antenna	95–102	≤ 4.8*	–	–	Medium	–
^29^	Series fed Array	98–103	13.4/12.2*	94	6.197 × 3.386 × 0.127	Low	–
^30^	Off-chip antenna	137–158	8.6*	81	12.3 × 4.5 × 0.905	Low	–
^31^	Photoconductive dipole antenna	1500	10.7	91.59	–	–	Sensing and imaging
This work	Planar antenna array (single layer)	100–110	14.35/13.9*	> 85	5.2 × 5.12 × 0.127 (1.82 λ_0_ × 1.79 λ_0_ × 0.044 λ_0_ at 105 GHz)	Low	Terahertz imaging and sensing

In response to these observed limitations, Our design aims to achieve an optimal balance between compactness, high efficiency and consistent performance. Our hybrid parallel-series fed array stands out by integrating a low-profile form with high gain and wide bandwidth. operating within the -10 db impedance bandwidth of 100–110 GHz achieves a peak gain of 14.35 dBi (simulated) and 13.9 dBi (measured). Furthermore, total efficiency exceeds 85% and exhibits less than 1 dBi variation in gain across the specified frequency range. Its compact size (5.2 × 5.12 × 0.127 mm) along with its classification is low-cost, making it an attractive option for standalone antenna applications.

## Conclusion

In this article, we propose a high-performance sub-THz antenna fabricated using low-cost PCB laser milling technology. The basic antenna design incorporates a quasi-slot integrated on a patch and is fed with a strip line. We have achieved an improved radiation pattern and higher antenna gain by modifying the radiation apertures. A 2 × 2 sub-THz antenna array was fabricated and tested, demonstrating a measured peak gain of 13.43 dBi at 105 GHz and a return loss better than 10 dB across the 100–110 GHz frequency range. The antenna was then optimized using machine learning-assisted global optimization techniques, which further improved the gain, bandwidth, and efficiency while minimizing the side lobe level within the desired band. The proposed antenna boasts a simple feeding structure, low fabrication costs, wide bandwidth, and high gain, making it well-suited for THz sensing and imaging applications.

## Data Availability

The datasets generated during and/or analyzed during the current study are available from the corresponding author on reasonable request.
