# Author Correction: A high-performance sub-THz planar antenna array for THz sensing and imaging applications

**DOI:** 10.1038/s41598-025-92482-y

**Published:** 2025-03-18

**Authors:** Muhammad Zubair, Abdul Jabbar, Farooq A. Tahir, Jalil ur Rehman Kazim, Masood Ur Rehman, Muhammad Imran, Bo Liu, Qammer H. Abbasi

**Affiliations:** 1 James Watt School of Engineering, University of Glasgow, Glasgow, G12 8QQ UK; 2 School of Electrical Engineering and Computer Science, National University of Sciences and Technology, Islamabad, Pakistan; 3 Artificial Intelligence Research Centre, Ajman University, Ajman, UAE

Correction to: *Scientific Reports* 10.1038/s41598-024-68010-9, published online 24 July 2024

The original version of this Article omitted an affiliation for Farooq A. Tahir. The correct affiliations are listed below.

James Watt School of Engineering, University of Glasgow, UK.

School of Electrical Engineering and Computer Science, National University of Sciences and Technology, Islamabad, Pakistan.

The original Article has been corrected.

